# Salt Tablets Safely Increase Serum Sodium in Hospitalised Elderly Patients With Hyponatraemia Secondary to Refractory Idiopathic Syndrome of Inappropriate Anti-Diuresis

**DOI:** 10.7759/cureus.24367

**Published:** 2022-04-22

**Authors:** Julia Calvo Latorre, Russell Senanayake, Waiel A Bashari

**Affiliations:** 1 Institute of Metabolic Science, Addenbrooke’s Hospital, Cambridge University Hospitals NHS Foundation Trust, Cambridge, GBR

**Keywords:** hospital discharge, fluid restriction, sodium chloride, siad, hyponatraemia

## Abstract

Hyponatraemia is the most common electrolyte abnormality encountered in the inpatient setting and is associated with increased morbidity, mortality, and length of hospital stay. Syndrome of inappropriate anti-diuresis (SIAD) remains the most common cause. Hyponatraemia due to SIAD presents various challenges in treatment approaches, including poor concordance (e.g., to fluid restriction), medication intolerance (e.g., demeclocycline), and risk of rapid sodium shifts (e.g., with vaptan therapy). The use of oral sodium chloride (NaCl) tablets is a recognised treatment approach. However, it is not commonly advocated. We present the cases of two elderly patients in whom the temporary use of NaCl tablets, as an adjunct to fluid restriction, led to safe and effective correction of SIAD-related hyponatraemia with resultant reduced length of hospital admission.

## Introduction

Hyponatraemia is the most common electrolyte abnormality encountered in clinical practice and is associated with increased morbidity and mortality [1,2]. It can present with a variety of symptoms depending on its onset and severity, including gait abnormalities, falls, and cognitive impairment [3,4]. Hospitalised patients with symptomatic hyponatraemia have a significantly higher mortality rate (20.6%), compared to those with normal serum sodium, and usually have prolonged hospitalisations [5].

Syndrome of inappropriate anti-diuresis (SIAD) is the most common underlying cause of hyponatraemia in the elderly population [6,7]. SIAD, which is a diagnosis of exclusion, is characterised by true hyponatraemia associated with a euvolemic body fluid status in the context of a urine osmolality greater than 100 mOsm/kg, detectable urine sodium (usually greater than 30 mmol/L), and with normal kidney, thyroid, and adrenocortical functions [8].

Following confirmation of SIAD, treating the cause (e.g., treating acute infections, stopping offending medications), together with a strict and supervised fluid restriction (FR), can result in the normalisation of serum sodium in the majority of cases [2]. The rate of serum sodium improvement should be gradual (8-10 mmol/24 hours) in most circumstances to avoid complications associated with rapid osmolar shifts (e.g., cerebral oedema and osmotic demyelination syndrome, ODS). Severe hyponatraemia (irrespective of the cause) may lead to neurological complications and requires prompt management with intense monitoring (e.g., in an intensive care unit) [9].

In idiopathic SIAD, which is more commonly observed in the elderly population [7], FR alone may not be effective [2], necessitating the introduction of pharmacological therapy. Treatment options include demeclocycline, lithium, urea, furosemide, or vasopressin-2-receptor antagonists. Demeclocycline and lithium can cause nephrotoxicity, whereas vasopressin-2-receptor antagonists can lead to excessively rapid correction of hyponatraemia with a high risk of developing ODS. Despite their use as second-line treatment options under the American guidelines, vaptans are not used enough in European countries due to the risk of overcorrection. Randomised controlled trials are needed to formally assess the benefit of using vaptans in this context. Furosemide increases the risks of concomitant hypokalaemia, and urea is not readily available in many countries.

The physiologic challenges in sodium homeostasis in the elderly population (which are usually due to frailty, concomitant use of offending medications, and kidney or cardiac impairment) present additional hurdles in SIAD diagnosis and management. Furthermore, the lack of symptoms in this cohort of patients, as a result of chronicity of hyponatraemia (e.g., in cases of reset osmostat), creates a false sense of reassurance among health professionals and may lead to unsafe discharge from the hospital with sodium levels that are too low to be safely managed in the community.

Sodium chloride (NaCl) tablets (alone or in conjunction with furosemide) have been used in treating acute-on-chronic SIAD-related hyponatraemia. However, there remains a paucity in the literature with regards to randomised controlled trials or first-line treatment studies in SIAD which result in a lack of standardisation of treatment in this condition. Here, we present the cases of two elderly patients in whom the temporary use of NaCl tablets as an adjunct to fluid restriction led to quick but safe correction of hyponatraemia with a resultant reduction in the duration of hospital stay.

## Case presentation

Case 1

Our first patient is a 74-year-old lady who was brought to the emergency department following an unwitnessed fall. Subsequent clinical assessment revealed a history of reduced balance, dizziness, and confusion for a few days. Relevant medical history included a recently treated urinary tract infection (UTI), polymyalgia rheumatica (with no regular steroid treatment), provoked pulmonary embolism following orthopaedic surgery, and a history of previous mild hyponatraemia (average serum sodium = 128 mmol/L). She was not taking any medications other than PRN paracetamol. Clinical examination only showed mild confusion with no other significant clinical findings. She weighed 77.4 kg and was not malnourished. She had euvolemic fluid status. Her serum sodium level on admission to our hospital was 121 mmol/L (reference range = 133-146 mmol/L).

Investigations confirmed true hyponatraemia and normal kidney, thyroid, and adrenocortical functions with an inappropriately raised urine osmolality (406 mmol/L) and urine sodium (71 mmol/L, Table 1). UTI was excluded. A brain computerised tomography (CT) scan showed old infarcts and age-related cerebral atrophy, while a CT of her chest revealed no radiological evidence of malignancy.

**Table 1:** Baseline biochemistry findings in both patients. FT4: free thyroxine; TSH: thyroid-stimulating hormone

Biochemical parameter (reference range)	Patient 1	Patient 2
Serum sodium (133–145) mmol/L	121	122
Serum osmolality (275–295) mmol/L	262	258
Serum urea (2.5–7.8) mmol/L	4.4	4.7
Serum creatinine (44–97) µmol/L	52	48
Serum TSH (0.35–5.5) mU/L	1.23	0.69
Serum FT4 (10.5–21) pmol/L	14.6	18
Serum cortisol (9 am) (>374) nmol/L	693	740
Urine osmolality (mOsm/kg)	406	455
Urine sodium (mmol/L)	71	32

**Figure 1:**
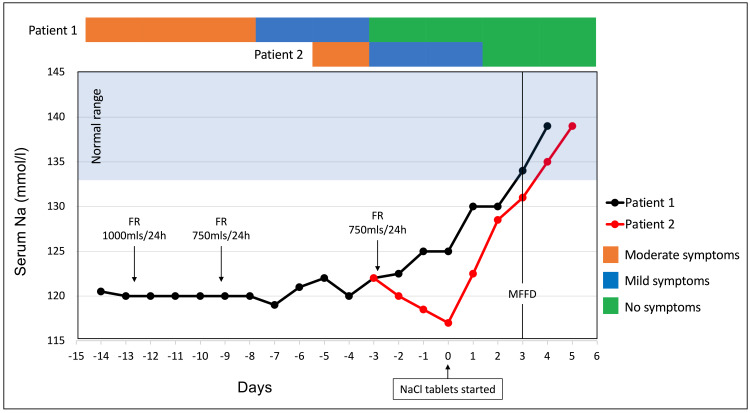
Sodium level and clinical course throughout the admission. FR: fluid restriction; MFFD: medically fit for discharge (achieved when patients’ sodium levels were ≥130 mmol/L); Na: sodium

Following a diagnosis of SIAD, 1 L over 24 hours of FR was started. Her serum sodium levels remained relatively static (119-121 mmol/L) despite FR (Figure 1). On day 14 of admission, she was transferred to the Endocrinology ward and her FR was strictly monitored at 750 mL per day. Her serum sodium levels improved slowly following this change. On day 19 of admission, due to the slow correction of her hyponatraemia, modified-release NaCl tablets at a dose of 2.4 g twice daily were initiated. At this point, her serum sodium was 125 mmol/L. Within 24 hours of NaCl commencement, the patient’s sodium level increased to 130 mmol/L accompanied by symptom resolution prompting discharge with a two-week course of NaCl tablets.

Follow-up by the patient’s local clinical team showed complete resolution of symptoms. A blood test for serum sodium, performed in the community, returned results in the normal range (139 mmol/L) with no additional treatment. Subsequent follow-up in the endocrine clinic was arranged. The cause of her SIAD was presumed secondary to a UTI.

Case 2

Our second patient is an 82-year-old lady with dizziness and abdominal pain who was transferred from a local hospital under the care of vascular surgeons for assessment and treatment of a large abdominal aortic aneurysm. Her medical history included a previous coronary artery bypass graft, multivalvular heart disease with a previous porcine aortic valve replacement, diet-controlled diabetes mellitus, hiatus hernia, and a previous left hip replacement. She was not taking any relevant medication at the time of transfer to our hospital. Clinical examination showed euvolemic fluid status with no other significant findings. She weighed 56.5 kg and did not appear malnourished. Routine investigations showed serum sodium of 122 mmol/L (reference range = 133-146 mmol/L), with a previous chronic mild hyponatraemia (average serum sodium = 130 mmol/L).

Again, true hyponatraemia was confirmed, together with normal kidney, thyroid, and adrenocortical functions. Urine examination showed inappropriately raised urine osmolality (455 mmol/L) and urinary spot sodium (32 mmol/L, Table 1). An abdominal CT confirmed the presence of a 6 cm abdominal aortic aneurysm. A chest X-ray and brain CT were unremarkable.

Initial treatment with intravenous 0.9% saline infusion was instigated. However, on her second day of admission and confirmation of SIAD, there was a discussion with the endocrine team with a recommendation for FR (750 mL in 24 hours). Subsequent deterioration of serum sodium, despite the resolution of her abdominal pain (which may have been the cause of her SIAD), prompted a tightening of FR (Figure 1). Modified-release NaCl tablets at a dose of 2.4 g twice daily were introduced on day three. Her hyponatraemia improved to 131 mmol/L within 72 hours and normalised within five days.

The patient’s aneurysm did not warrant surgical intervention due to the increased risks associated with surgery in the context of significant co-morbidities. She was deemed fit for discharge from the hospital, following which a sustained normal serum sodium level was demonstrated. Unfortunately, she died soon afterwards due to a major cardiac event. Fluid status remained normal with no evidence of heart failure. The cause of her SIAD was never fully established but might have been secondary to abdominal pain.

## Discussion

We report the cases of two hospitalised elderly patients with profound symptomatic hyponatraemia secondary to idiopathic SIAD where the addition of NaCl tablets as an adjunct to ongoing FR prompted discharge and sustained sodium levels post-discharge. Both patients had clinical and biochemical confirmation of SIAD in the context of a chronic history of mild hyponatraemia (at least a year prior to admission), which makes an occult malignant aetiology very unlikely.

Once SIAD is confirmed, treating the underlying cause and instigating a closely monitored FR protocol is the most important step in the treatment process [2]. Lack of supervision may occasionally lead to inadvertent interruption of FR, particularly in cases of elderly patients with primary (or hyponatraemia-induced) cognitive impairment. In such scenarios, where there has been slow improvement in serum sodium, reassessment (clinical and biochemical) is essential with reinforcement of strict FR if SIAD remains the likely cause. In case of a slow-to-improve (or deteriorating) serum sodium despite accurate FR, reassessment is warranted to ensure the correct diagnosis is made. If SIAD remains the likely disease process, the introduction of pharmacological agents for SIAD treatment is warranted.

**Figure 2:**
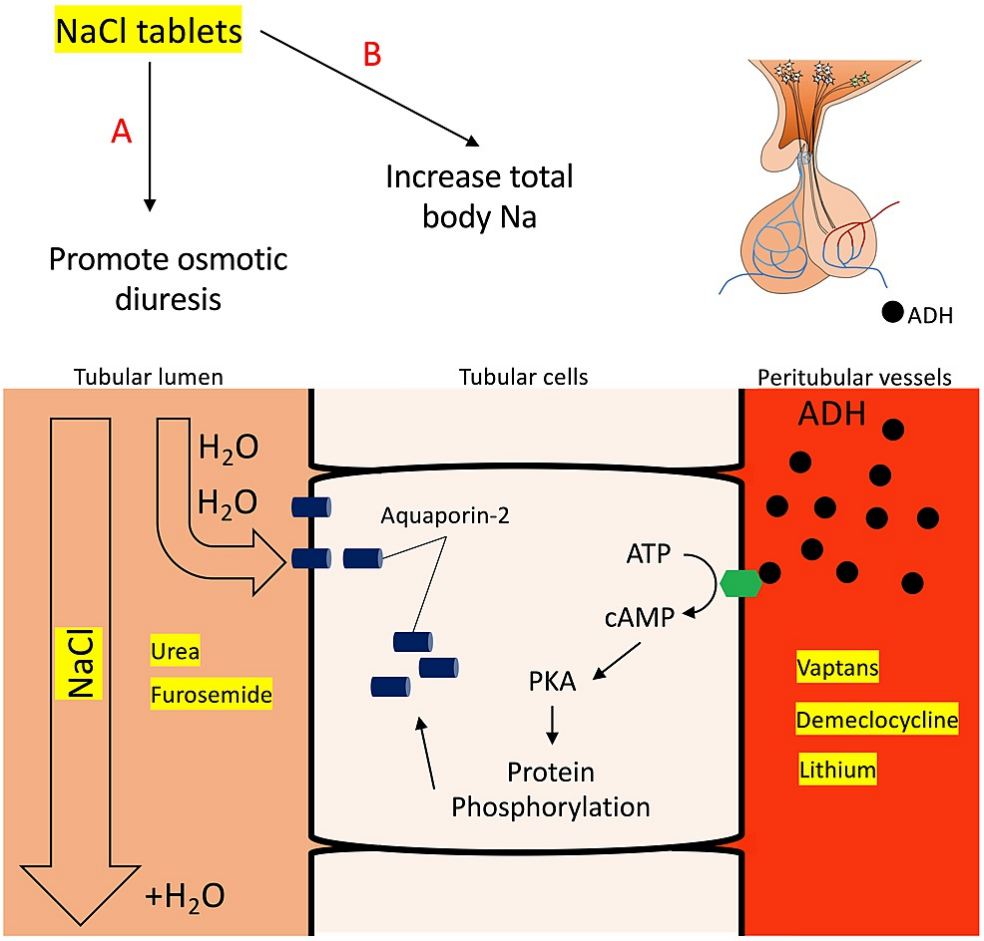
Pharmacological treatment in SIAD. Vaptans, demeclocycline, and lithium act by blocking/reducing the effect of ADH on its receptor at the renal tubular cells’ basal membrane, whereas urea, furosemide, and NaCl tablets increase water excretion by promoting osmotic diuresis. ADH: antidiuretic hormone; ATP: adenosine triphosphate; cAMP: cyclic adenosine monophosphate; NaCl: sodium chloride; PKA: protein kinase A

Vaptans are potent vasopressin receptor antagonists associated with serious concerns of rapid correction of hyponatraemia and require close monitoring of serum sodium to avoid ODS. Hepatotoxicity is another concern and may further limit their use [10]. Demeclocycline and lithium act by diminishing the responsiveness of the antidiuretic hormone receptor in the collecting ducts (thus producing a drug-induced nephrogenic diabetes insipidus) (Figure 2). Unfortunately, these agents can also cause significant side effects and are not advocated as first-line pharmacological therapy in SIAD. Urea, which acts by promoting osmotic diuresis as well as sodium retention in the renal tubule, can be poorly tolerated and is not readily available in many countries.

NaCl tablets have been administered concurrently with loop diuretics in the treatment of SIAD. The latter work by inhibiting the reabsorption of sodium and chloride in the loop of Henle, thus reducing tonicity in the interstitium and increasing the excretion of free water. Some trials recommend the use of NaCl tablets together with loop diuretics as a second-line treatment for the management of SIAD [11]. However, in doing so, they also promote the excretion of extra sodium in the urine and reduce the total sodium level in the body. Moreover, the addition of furosemide may lead to the development of hypokalaemia and renal impairment, particularly with prolonged use. In our patients, NaCl tablets were used as a single drug agent together with fluid restriction with good outcomes. Furthermore, the benefit was sustained for several weeks following discharge from the hospital in the first patient.

In patients with prolonged SIAD-induced hyponatraemia, where inappropriate renal sodium excretion had occurred for several days to weeks, it is reasonable to assume that these patients have eventually developed a reduced total body sodium state which would contribute to the slow recovery of hyponatraemia (i.e., becoming salt deficient) [12]. Therefore, a diet with high sodium content may improve this possibility; elderly patients are unlikely to tolerate such a diet while admitted with symptomatic hyponatraemia. Modified-release NaCl tablets offer high sodium delivery within a small tablet that is easy to tolerate. Furthermore, NaCl tablets in this context may promote the excretion of water by increasing osmotic diuresis and thus drive water out of the tubular cells into the renal tubules (Figure 2).

The association between higher NaCl intake and hypertension has been widely reported with links to worse cardiovascular outcomes. Therefore, it is advised that NaCl tablets should only be used temporarily in refractory SIAD to facilitate discharge from the hospital until the underlying cause for SIAD is fully elucidated in an outpatient setting and a safe management plan is developed.

## Conclusions

Hyponatraemia remains the most common electrolyte imbalance. It is particularly problematic in hospitalised elderly patients where it is associated with increased morbidity and mortality and, therefore, should not be ignored. While SIAD remains the most common cause of hyponatraemia in the elderly, accurate and prompt identification of the cause is essential in the management process. Strict and closely supervised FR is the first-line therapy in hyponatraemia caused by SIAD. FR refractory SIAD results in prolongation of hospital stay and should prompt the use of pharmacological agents, with careful supervision. The temporary use of oral NaCl tablets, in addition to FR, is a safe option in the treatment of refractory SIAD in the elderly in whom a degree of solute depletion is suspected.
